# *Salmonella* Persistence in Infection: Molecular Regulation, Host Microenvironments, and Multiscale Heterogeneity

**DOI:** 10.3390/microorganisms14051073

**Published:** 2026-05-09

**Authors:** Dandan Ding, Hui Sun, Jing Yang

**Affiliations:** Cuiying Biomedical Research Center, The Second Hospital & Clinical Medical School, Lanzhou University, Lanzhou 730030, China; dingdd2023@lzu.edu.cn (D.D.); suihun@lzu.edu.cn (H.S.)

**Keywords:** *Salmonella*, persistence, persister cells, antibiotic tolerance, host microenvironment, stress-response networks, infection relapse, antibiotic resistance

## Abstract

*Salmonella* persistence contributes to infection relapse, chronic carriage, and reduced antibiotic efficacy. Traditionally viewed as dormant subpopulations that passively survive antibiotic exposure, persister cells are now increasingly recognized as dynamic, heterogeneous, and context-dependent physiological states shaped by bacterial regulatory programs and host microenvironmental pressures. This review examines *Salmonella* persistence from a multiscale perspective. We first clarify key antibiotic survival phenotypes, including resistance, heteroresistance, tolerance, persistence, and viable but non-culturable states. We then discuss how host-derived stressors, such as phagosomal acidification, nutritional restriction, metal perturbation, and reactive oxygen and nitrogen species, promote growth-restricted, persistence-associated bacterial states. At the bacterial level, we summarize stress-response networks involving the stringent response, SOS response, toxin–antitoxin systems, and auxiliary regulators that coordinate metabolic remodeling, growth restriction, and antibiotic survival. At the host level, we highlight how organ reservoirs, immune cell subsets, metabolic cues, and *Salmonella*-mediated immune niche remodeling shape persistence-associated phenotypes in vivo. Finally, we discuss clinical and translational implications, including endogenous relapse, resistance evolution, and emerging anti-persistence strategies. Together, this review provides a framework for understanding *Salmonella* persistence as a multiscale, niche-dependent process relevant to recurrent and chronic infection.

## 1. Introduction

*Salmonella enterica* is one of the most significant global foodborne pathogens, classified within the family *Enterobacteriaceae*. It is a Gram-negative, facultative intracellular pathogen with over 2600 identified serotypes. These serotypes are primarily classified into typhoidal *Salmonella* and non-typhoidal *Salmonella* (NTS) [1,2]. *Salmonella* is transmitted to healthy hosts through the consumption of contaminated food and water [1]. Typhoidal and non-typhoidal *Salmonella* infections together impose a substantial global disease burden, affecting millions of people each year. Meanwhile, the rising antibiotic resistance has become an increasing concern. Data from the U.S. Centers for Disease Control and Prevention (CDC) indicate that between 2004 and 2016, the incidence of NTS infections with clinically significant antibiotic resistance increased by 40% [3].

*Salmonella* infections are associated with significant morbidity and mortality, particularly in invasive cases. A global meta-analysis revealed that the case-fatality rate for invasive NTS infections is as high as 14.7%, with frequent complications, and the disease burden is largely concentrated in Africa and Asia [4].

In addition to recurrence following acute infection, *Salmonella* can also establish chronic asymptomatic carriage. Chronic carriers of typhoidal *Salmonella* serve as important reservoirs for pathogen maintenance and transmission, with the gallbladder and bile ducts acting as key sites for long-term colonization [5,6]. During infection, a subpopulation of *Salmonella* enters a reversible, non-proliferative state known as persister cells. These cells survive lethal antibiotic exposure without acquiring genetic resistance, often by residing within protected intracellular niches. Upon stress removal, persisters can resume growth, contributing to infection relapse and chronicity [7].

As a facultative intracellular pathogen, *Salmonella* provides a valuable model for studying bacterial persistence in the context of complex host–pathogen interactions. While persister cells were traditionally viewed as passive dormant subpopulations, recent single-cell studies in infection models suggest that persistence-associated states may also involve active adaptation to the host–cell environment, accompanied by distinct physiological and virulence-related features [8]. In persistent infections, typhoidal and non-typhoidal *Salmonella* exhibit different clinical manifestations. Typhoidal *Salmonella* is often associated with asymptomatic carriage, whereas non-typhoidal *Salmonella* more commonly causes symptomatic persistent infections, although both can function as reservoirs for long-term infection [9].

Persister cells represent a subpopulation of bacteria capable of surviving high concentrations of antibiotics without undergoing genomic changes. Unlike traditional antibiotic resistance acquired through genomic alterations, persister cells evade antibiotic killing by entering a reversible non-proliferative or slow-proliferative state. Once the stress is alleviated, they can resume growth, becoming a central factor in infection recurrence and chronicity [10,11]. Bacterial populations exhibit more complex survival strategies under antibiotic pressure, including tolerance and persistence. Tolerance extends bacterial survival by slowing metabolism at the population level. Persistence, in contrast, allows a small subpopulation to enter a growth-arrested state to evade antibiotic killing, with growth resuming once the stress is removed [12,13].

Recent advances in antibiotic persistence research have provided an important foundation for understanding *Salmonella* recurrence and chronic infection. Earlier reviews emphasized *Salmonella* long-term persistence and transmission within the host, particularly highlighting the role of host colonization dynamics and the gut microbiota in controlling infection outcomes [14]. Subsequently, the 2019 consensus statement on antibiotic persistence established a unified conceptual and methodological framework for the field by clearly distinguishing persistence from resistance and tolerance and introducing key parameters such as minimum inhibitory concentration (MIC) and minimum duration of killing (MDK) [7]. More recently, Giorgio and Helaine reviewed the in vivo progression of *Salmonella* antibiotic tolerance, providing important insights into bacterial survival strategies during infection and their relevance to recurrence [15]. Together, these studies have significantly advanced the field, with each contributing valuable insights into conceptual definitions, host colonization, or in vivo tolerance progression.

Building on this, the present review attempts to examine the biology of *Salmonella* persistence from a multiscale perspective that integrates intracellular regulatory mechanisms with tissue-specific host microenvironments, immune niche remodeling, and potential intervention strategies. One aspect worth further exploration is how host tissue and cellular heterogeneity shape persistence formation, maintenance, and reactivation across different anatomical sites. In this context, we further discuss how *Salmonella* actively reshapes immune niches during persistent infection and summarize emerging therapeutic strategies targeting persisters, including metabolic awakening, immune modulation, and ecological interventions. Through an integration of molecular mechanisms, spatial ecology, and translational implications, this review offers a framework for considering *Salmonella* persistence and its clinical consequences.

## 2. Conceptual Framework of Antibiotic Survival Phenotypes in *Salmonella*

Treatment failure and relapse in *Salmonella* infections are multifactorial. The ability of bacteria to survive under both antibiotic exposure and host immune pressure represents a key contributing factor. This survival capacity cannot be attributed to a single mechanism but instead reflects a spectrum of genetically encoded resistance and non-genetic survival strategies operating at both the population and single-cell levels [16].

Building on the conceptual framework established by the 2019 consensus on antibiotic persistence [7], bacterial survival under antibiotic stress is commonly described in terms of several related but distinct phenomena, including resistance, heteroresistance, tolerance, and persistence. Within this framework, resistance refers to stable, heritable traits that enable bacterial growth in the presence of antibiotics, whereas tolerance and persistence primarily reflect non-genetic phenotypic adaptations associated with altered killing dynamics. Heteroresistance, in contrast, captures population-level heterogeneity in antibiotic susceptibility within an isogenic bacterial population.

Based on this framework, this section systematically outlines these categories and their manifestations in *Salmonella* infections, providing a foundation for the subsequent discussion. Table 1 summarizes the key distinctions among resistance, heteroresistance, tolerance, persistence, and viable but non-culturable (VBNC) states.

**Table 1:** Conceptual distinctions among resistance, heteroresistance, tolerance, persistence, and VBNC states in *Salmonella*.

Phenotype	Definition	Key Measurement Parameters	Heritability	Reversibility	Typical Infection-Context Manifestation	Examples in *Salmonella*	References
Resistance	Growth in the presence of antibiotics	Increased MIC	Genetic (mutation, HGT)	Stable	Bacteria continue to grow during treatment; treatment failure	β-lactamase carried on plasmids; gyrase mutations	[10,17]
Heteroresistance	A subpopulation grows at antibiotic concentrations higher than the MIC of the main population	Increased MIC in a subpopulation; typically requires PAP analysis	Usually associated with unstable gene amplification or other non-fixed genomic changes	Variable	Appearance of resistant subclones during treatment	Unstable gene amplification in clinical isolates of *Salmonella* Typhimurium	[18,19]
Tolerance	The population survives antibiotic exposure without sustained growth	Increased MDK at the population level	Physiological (non-genetic)	Reversible	Slower bacterial clearance; MIC unchanged	Slow growth due to nutrient limitation in granulomas	[12,20]
Persistence	A small subpopulation survives antibiotic exposure without sustained growth	Increased MDK in a subpopulation; typically associated with a biphasic kill curve	Non-genetic; often stochastic or epigenetic in origin	Reversible	Infection relapses after cessation of antibiotics; rare cells surviving under high concentrations of antibiotics	Persister formation in macrophages associated with the stringent response	[7,16,21,22,23,24]
VBNC	Metabolically active but unable to grow on standard media	Loss of culturability	Non-genetic	Reversible under specific conditions	Long-term survival in environmental reservoirs; undetectable by traditional culture methods	*Salmonella* in biofilms under low-temperature storage; *Salmonella* Typhi induced by CuSO_4_	[25,26,27,28]

### 2.1. Resistance, Heteroresistance, Tolerance, and Persistence

Bacterial phenotypes that enable survival despite antibiotic exposure can be differentiated based on their effects on two key parameters: MIC and MDK. Resistance and heteroresistance are characterized by an increased MIC, whereas tolerance and persistence are defined by an extended MDK without a change in MIC [7]. Resistance enables the entire population to grow in the presence of antibiotics, typically via mutations or horizontal gene transfer (HGT) [10]. Resistance in *Salmonella* is becoming increasingly severe, particularly in sub-Saharan Africa, where approximately 75% of *Salmonella enterica* Typhi isolates exhibit multidrug resistance [17]. Heteroresistance refers to a small subpopulation exhibiting an MIC significantly higher than the rest (typically >8-fold), often associated with gene amplification [18,19]. Tolerance is a population-level phenotype that prolongs survival via slow growth, without changes in MIC [12,20]. Persistence affects a small subpopulation, exhibiting a biphasic kill curve [21], and is reversible [7,16,23].

### 2.2. Persister Cells: Definitions, Types, and In Vivo Manifestations

Throughout this review, persister cells refer to a small, transient subpopulation of genetically susceptible bacteria that survive antibiotic exposure without acquiring heritable resistance. These cells enter a reversible non-growing or extremely slow-growing state under antibiotic or host-derived stresses and can resume proliferation upon stress removal [7,16].

Persister cells are conceptually distinct from both dormancy and tolerance. Dormancy represents a broader non-proliferative physiological state, whereas persistence specifically refers to the ability to survive lethal antibiotic exposure [7]. Importantly, not all dormant cells exhibit a persister phenotype; for example, stationary-phase bacteria are typically non-growing but do not necessarily display antibiotic tolerance associated with persistence [7]. In contrast, tolerance describes a population-wide reduction in growth rate that prolongs survival without altering the minimum inhibitory concentration (MIC) [12,21].

**Figure 1:**
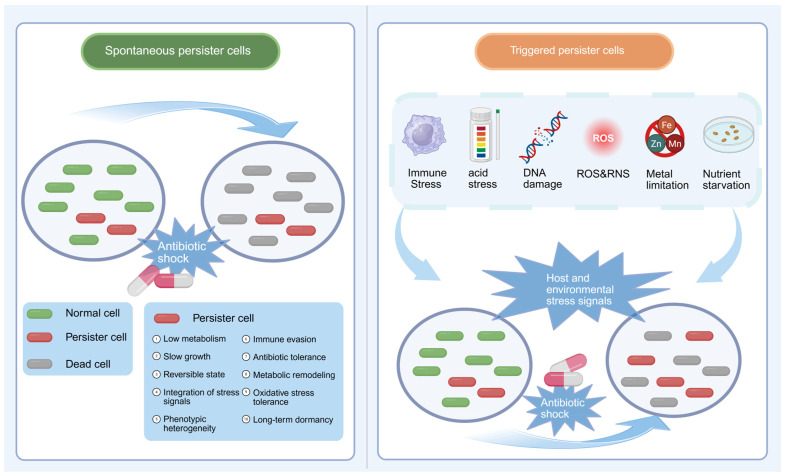
Operational classification of spontaneous and induced persister cells. (**Left**) Spontaneous persisters arise stochastically without overt external stress. (**Right**) Induced persisters emerge in response to host- or environment-derived stressors, integrated through bacterial stress-response networks. BioRender Inc. (Toronto, ON, Canada). https://BioRender.com (accessed on 22 February 2026).

Based on the consensus framework [7], persisters can be broadly classified into spontaneous and induced types (Figure 1). Spontaneous persisters arise stochastically in the absence of external stress, reflecting intrinsic phenotypic heterogeneity within bacterial populations [29]. Induced persisters, in contrast, emerge in response to environmental cues, particularly host-associated stresses encountered during infection, such as nutrient limitation, acidification, and immune-mediated pressures [29,30]. These signals are integrated into bacterial stress-response networks (see Section 4), leading to a reversible low-metabolic state that enhances survival under adverse conditions [31,32]. Notably, exit from the persister state is also heterogeneous, and a subset of cells may remain growth-arrested even after stress removal [12].

In vivo, *Salmonella* persistence is shaped by complex host microenvironments. Host-derived stresses, including acidic pH, nutrient restriction, reactive oxygen species (ROS), and reactive nitrogen species (RNS), drive bacteria into growth-restricted states [29,33]. Within infected tissues, at least two survival patterns can coexist [21,22]: (i) a population-wide reduction in growth rate consistent with antibiotic tolerance, characterized by uniformly decreased metabolic activity without changes in MIC [20]; (ii) a smaller subpopulation entering a deeper dormant state via stochastic or stress-induced mechanisms, consistent with classical persisters [29].

Single-cell analyses have provided direct evidence for non-replicating intracellular bacteria that can resume proliferation after stress relief, with progeny retaining antibiotic susceptibility [29]. In addition, *Salmonella* can exploit specific host niches, such as iron-rich erythrophagocytic macrophages, to alleviate nutritional constraints and support persistence [30]. The relative contributions of tolerance-like and persister-like states vary depending on tissue niche, immune context, and stage of infection [20,30,33]. For example, in zebrafish infection models, early M1-like macrophages contribute to bacterial control, whereas later M2-like macrophage populations provide a more permissive environment for persistence [33]. Consistently, Fanous et al. found that under certain conditions, population-wide slow growth may dominate treatment failure, although classical persisters remain detectable [24,34].

## 3. Host-Derived Stressors Shaping Persistence-Associated States

In mouse and macrophage infection models, *Salmonella* can adopt non-replicating intracellular states following phagocytic uptake. These observations are consistent with the idea that host-associated immune and metabolic stresses contribute to the emergence of growth-restricted, persistence-associated states under experimental conditions [24,35]. Importantly, current evidence does not support a simple model in which host immunity directly and uniformly “induces” persistence. Rather, it suggests that multiple host-derived factors, including antimicrobial effector mechanisms, nutrient limitation, and metabolic constraints, collectively shape bacterial physiological states and promote the emergence or maintenance of persistence-associated subpopulations in specific niches. In certain contexts, such states may contribute to post-treatment bacterial survival and recurrent infection, although direct causal links between persistence and relapse in human *Salmonella* infections remain incompletely established [35] (Figure 1).

### 3.1. Phagosomal Acidification and Acid-Stress Adaptation

The phagosomal microenvironment established following *Salmonella* uptake provides a key intracellular context in which persistence-associated states may arise. Upon internalization, bacteria are rapidly exposed to a combination of hostile conditions, including vacuolar acidification, disrupted ion homeostasis, and severe nutrient limitation [36]. Among these stressors, acid stress represents a major challenge encountered by *Salmonella* both within the phagosome and along the gastrointestinal tract. *Salmonella* senses acidic pH through the EnvZ/OmpR two-component system, which coordinates adaptive responses that enhance acid resistance and modulate virulence gene expression [36,37]. Global transcriptomic analyses indicate that the acid tolerance response of *Salmonella* involves extensive reprogramming of bacterial gene expression, affecting hundreds of genes. These changes are associated with reduced energy expenditure, activation of DNA repair and oxidative damage repair systems, and maintenance of intracellular pH homeostasis. This pH balance is supported by metabolic adjustments, including modulation of the NAD^+^/NADH ratio and remodeling of tricarboxylic acid (TCA) cycle activity [38]. This metabolic remodeling may push a subset of the bacterial population toward a non-replicating or slow-growing state, consistent with the emergence of a persistence-associated subpopulation with elevated antibiotic survival in macrophage infection models [24].

### 3.2. Nutritional Immunity and Metabolic Restriction

In addition to acid stress, macrophages exert selective pressure through nutritional immunity. The host limits the availability of essential metal ions, such as iron (Fe), zinc (Zn), and manganese (Mn), or accumulates antimicrobial concentrations of copper (Cu) [39]. These metal perturbations constrain core bacterial metabolic processes, including processes linked to respiration, DNA maintenance, and antioxidant defense that depend on metal cofactors, and may thereby favor growth-restricted states under intracellular stress conditions [40] (Figure 1). To cope with the combined challenge of metal starvation and intoxication, *Salmonella* finely regulates its metal ion uptake, storage, and efflux systems [39], which may increase metabolic burden, slow growth, and favor the emergence of persistence-associated physiological states.

The limitation of key metabolic resources may also influence the likelihood of entering growth-restricted states associated with persistence. For example, arginine metabolism contributes to intracellular pH homeostasis and antioxidant defense. Under oxidative stress, arginine-biosynthesis-deficient strains (ΔargCBH) show reduced virulence and increased sensitivity to hydrogen peroxide, a phenotype associated with the collapse of intracellular ΔpH and impaired antioxidant capacity [41]. Although these findings do not directly demonstrate persister formation, they support the broader idea that metabolic insufficiency can reshape bacterial stress adaptation and modulate the probability of entry into survival-associated low-growth states.

### 3.3. ROS/RNS-Mediated Growth Restriction and Reactivation

In addition to nutrient limitation, ROS and RNS produced by immune cells, such as macrophages, serve as critical stress signals that may promote the entry of bacterial subpopulations into persistence-associated states. In murine macrophage infection models, ROS derived from macrophages have been shown to induce the formation of intracellular protein aggregates (aggresomes) in *Salmonella*. Bacteria containing these aggregates exhibit features consistent with a dormancy-associated or deeply growth-restricted state, including reduced ATP levels, suppressed metabolic activity, and elevated antibiotic survival, consistent with a persistence-associated phenotype. More importantly, upon stress relief, these cells were reported in that model to restore virulence programs associated with *Salmonella* pathogenicity island 1 (SPI-1) and SPI-2, and resume proliferation [42]. These findings suggest that ROS-mediated protein aggregation may be linked to entry into persistence-associated states and prolonged intracellular survival, although its contribution to clinical recurrence remains inferential and should be interpreted cautiously.

Nitric oxide (NO) and its derivatives are key effector molecules in the host’s innate immune response to *Salmonella* [43]. In later stages of infection, RNS may contribute to maintaining subpopulations of intracellular bacteria in growth-restricted states, including cells with persistence-associated features. One proposed mechanism is that NO inhibits key enzymes in the TCA cycle, thereby reducing succinyl-CoA availability and constraining amino acid biosynthesis and cellular energy metabolism [44]. Notably, this “metabolic lock” appears reversible: when host immune pressure decreases and RNS levels decline, growth-restricted bacteria gradually restore metabolic activity and resume heterogeneous outgrowth [45]. This reversibility is consistent with a potential role in post-stress survival and may, in principle, contribute to relapse, although direct evidence in human infection remains limited.

In summary, the host stress signals *Salmonella* encounters—acid stress, metal ion starvation/toxicity, nutritional restriction, and ROS/RNS—converge on a common physiological outcome: reduced intracellular ATP levels and slowed bacterial growth. Reduced ATP and slow growth are widely associated with persistence-associated (or persister-like) physiology [24,46] and are considered to contribute to both population-level tolerance and subpopulation-level persistence [12,47]. Thus, growth rate and energetic state are proposed as key physiological intermediates linking host-derived stresses to persistence-associated phenotypes.

## 4. Bacterial Regulatory Networks Underlying *Salmonella* Persistence

**Figure 2:**
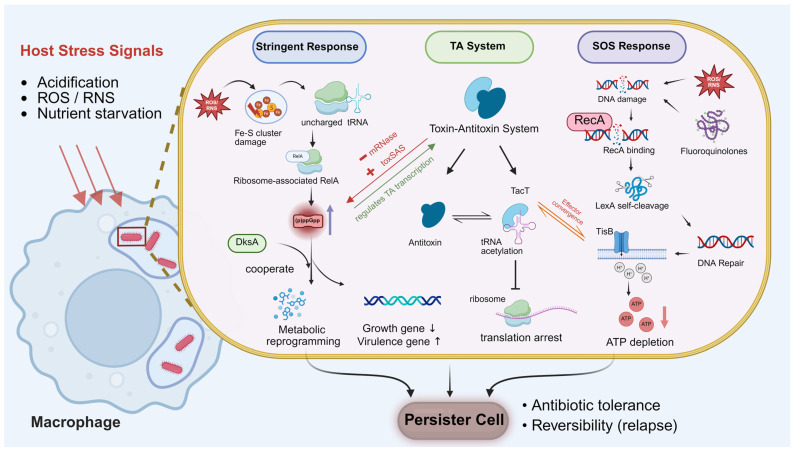
Integrated bacterial stress-response networks contributing to persistence-associated states. The schematic illustrates three major stress-response pathways that promote bacterial persistence under host-derived stress conditions, including acidification, reactive oxygen/nitrogen species (ROS/RNS), and nutrient limitation. Stringent response): RelA/SpoT synthesize guanosine tetra-/pentaphosphate ((p)ppGpp), which, together with DksA, reprograms transcription and promotes metabolic adaptation. TA system: Stress-induced degradation of antitoxins releases toxins such as TacT, which acetylates tRNA and inhibits translation, leading to growth arrest. SOS response: DNA damage activates RecA, triggering LexA self-cleavage and induction of SOS genes; the TisB toxin disrupts membrane potential, causing ATP depletion and growth arrest. Collectively, these pathways contribute to a reversible persister state characterized by reduced metabolic activity, antibiotic tolerance, and the potential for relapse. Abbreviations: (p)ppGpp, guanosine tetra-/pentaphosphate; ROS, reactive oxygen species; RNS, reactive nitrogen species; toxSAS, toxin small alarmone synthetase; mRNase, mRNA interferase; TacT, tRNA acetyltransferase toxin; TisB, membrane pore-forming toxin. BioRender Inc. (Toronto, ON, Canada). https://BioRender.com (accessed on 23 March 2026).

During systemic or intracellular stages of infection, *Salmonella* frequently occupies macrophages and other host-associated intracellular niches. After engulfment, the bacteria are exposed to various antimicrobial stressors, including phagosomal acidification, ROS, RNS, and nutritional restriction [39]. These stresses can engage the stringent response, SOS response, and toxin–antitoxin (TA) systems, although the relative contribution of each pathway likely depends on the niche and stress context [40]. (Figure 2).

### 4.1. The Stringent Response and Persistence-Associated Growth Restriction

*Salmonella* integrates multiple host-imposed stress signals through the stringent response, which is widely implicated in the emergence and maintenance of persistence-associated states. The core of the stringent response is the accumulation of intracellular signaling molecules, guanosine tetraphosphate and guanosine pentaphosphate ((p)ppGpp), whose levels are co-regulated by RelA and SpoT. RelA primarily responds to ribosome stalling induced by amino acid starvation. SpoT senses various signals, such as fatty acid limitation, phosphate deficiency, and oxidative stress. Through its bifunctional synthesis and hydrolysis activity, SpoT maintains the dynamic balance of (p)ppGpp [48] (Figure 2).

During infection, host-associated nutrient limitation and immune-derived stresses are important triggers of the stringent response, although the dominant signal likely varies across intracellular and tissue contexts [49]. Recent studies have revealed a pathway by which host oxidative stress activates the stringent response: within macrophages, ROS and RNS can cause bacterial iron–sulfur cluster damage, limit de novo synthesis of sulfur-containing and branched-chain amino acids, trigger RelA activation at the ribosome, and lead to marked (p)ppGpp accumulation [50] (Figure 2).

The accumulation of (p)ppGpp triggers global metabolic remodeling and is widely considered to create a physiological context favorable for entry into persistence-associated states. By downregulating genes associated with macromolecule synthesis and cell division, while upregulating genes related to stress adaptation and virulence, (p)ppGpp may shift *Salmonella* toward a low-metabolism, persistence-associated state [49]. Under in vitro conditions, intracellular (p)ppGpp accumulation induced by nutritional limitation or antibiotic stress has been associated with increased entry into low-metabolism, persistence-associated states [12].

This metabolic remodeling is not solely mediated by (p)ppGpp and, in many contexts, also involves the transcriptional regulator DksA. As an essential transcriptional regulator in the stringent response, DksA modulates transcription initiation by binding to the secondary channel of RNA polymerase, acting together with (p)ppGpp, and regulates glutathione synthesis and the pentose phosphate pathway to maintain intracellular redox homeostasis [51]. The function of DksA is condition-dependent: under certain stresses, DksA is required to enhance the function of (p)ppGpp, while under other conditions, alternative factors may compensate [52]. Moreover, ROS and RNS, as primary stressors imposed by the host, require DksA-mediated antioxidant and anti-nitrosative defenses to ensure bacterial survival. This process depends on the cooperation between DksA and (p)ppGpp [51,53] (Figure 2).

In murine macrophage infection models, single-cell analysis has demonstrated that *Salmonella* within macrophages can form a non-replicating persister subpopulation, and this process has been linked to the stringent response [24]. Studies have shown that the deletion of *dksA* significantly reduced bacterial survival within macrophages, and its sensitivity to NO could be reversed by amino acid supplementation, suggesting an intrinsic link between amino acid metabolism, oxidative defense, and the formation of persistence-associated states [53]. In a mouse infection model, *Salmonella* Typhimurium mutants lacking *relA* and *spoT* completely lost their ability to systemically disseminate and cause lethality [54]. *DksA* deletion strains, on the other hand, exhibited severe colonization defects in both intestinal and systemic infections [52]. Notably, the virulence of these strains was restored in inducible nitric oxide synthase (iNOS) knockout mice (lacking endogenous NO production) [53].

The stringent response-regulated dormancy program is not limited to classical persister cells. In *Bacillus subtilis*, (p)ppGpp promotes the formation of persister cells by lowering GTP levels [55], suggesting that nucleotide metabolism reprogramming is a broadly conserved mechanism for bacterial growth restriction and persistence-associated physiology.

### 4.2. The SOS Response in Persistence-Associated Survival and Resistance Evolution

The SOS response is an inducible DNA damage repair system that plays a central role in bacterial responses to genotoxic stress. The RecA protein binds to single-stranded DNA and induces the autocleavage of the transcriptional repressor LexA, thereby initiating the expression of DNA repair genes [56]. In various bacterial pathogens, the SOS response has been implicated in the emergence of persistence-associated states [57] and is also closely linked to the evolution of antibiotic resistance [58]. The chemical inhibitor targeting RecA, BRITE-338733, has been reported to reduce *Salmonella* antibiotic tolerance under experimental conditions [59] (Figure 2).

The contribution of the SOS response to persistence may involve at least two aspects: first, the repair of DNA damage, which may support bacterial survival under stress; and second, the induction of mutagenic pathways, which may increase the potential for subsequent genetic adaptation [58]. In this sense, persistence-associated states may provide a physiological context that facilitates, rather than directly causes, the evolution of genetic resistance.

The contribution of the SOS response to the emergence of persistence-associated states has primarily been inferred from in vitro antibiotic stress studies, with limited direct in vivo evidence. In experimental models, fluoroquinolone exposure induces DNA damage and activates the SOS response, which is linked to the induction of toxin–antitoxin modules and the establishment of growth-arrested, low-energy states that promote phenotypic antibiotic survival, features consistent with persistence-associated phenotypes [57] (Figure 2). The persister cells induced by different antibiotics exhibit differential transcriptional profiles: fluoroquinolones primarily activate the SOS response and related toxin genes (e.g., *recA*, *tisB*, *sulA*), whereas β-lactam antibiotics more profoundly affect bacterial metabolism and motility pathways [60]. This suggests that the mechanisms underlying persistence formation are stressor-specific.

It remains unclear whether activation of the SOS response directly contributes to persister cell formation during macrophage infection. Although reactive oxygen and nitrogen species produced by macrophages can cause bacterial DNA damage, whether this damage triggers SOS-dependent persister formation in the intracellular environment has not been directly demonstrated [42,44]. RecA, a central regulator of the SOS response, is positioned at the interface between DNA-damage signaling and persister-related outcomes. Current evidence suggests two roles for RecA in the context of persister biology. RecA may support the survival of intracellular persisters by repairing host-imposed DNA damage. In parallel, RecA-mediated activation of the SOS response contributes to antibiotic tolerance in *Salmonella*, and its pharmacological inhibition reduces tolerance [59]. However, whether RecA directly contributes to persister cell formation within the intracellular environment remains to be established.

### 4.3. Toxin–Antitoxin Systems as Context-Dependent Modulators of Growth Restriction

TA systems are widely present in bacterial genomes and typically consist of stable toxins and unstable antitoxins. Toxins induce growth arrest by inhibiting DNA replication, translation, or cell wall synthesis, while antitoxins neutralize toxin activity [61]. Under steady-state conditions, antitoxins suppress toxins; upon stress, antitoxins may be destabilized, allowing toxin activity to promote reversible growth restriction and the emergence of persistence-associated states [62] (Figure 2).

Multiple TA systems are transcriptionally upregulated by antibiotic or host immune stress [62], but their contribution to persister cell formation remains contentious. Early studies suggested functional redundancy among TA modules in promoting persistence [63]; yet, recent reviews question this, noting that removal of all type II TA systems in *E. coli* did not affect persister levels [61]. TA systems may instead be involved in mobile genetic element maintenance or viral defense. Single-cell studies indicate that TA effects are stochastic and strongly condition-dependent [63].

In *Salmonella*, among six conserved TA loci (five RelBE family, one VapBC family), deletion of specific loci alters stress tolerance in a stress-type-dependent manner. Notably, specific toxins are induced within macrophages, suggesting selective activation by the host intracellular environment [64]. For example, the acetyltransferase toxin TacT is induced during macrophage infection, where it acetylates tRNA to block translation elongation, thereby promoting growth arrest. This process is reversible via a specific peptidyl-tRNA hydrolase, allowing recovery upon stress relief [65]. *Salmonella* may also use multiple toxins (e.g., TacT and TacAT2) in combination to cumulatively inhibit different tRNA subgroups, prolonging intracellular survival [66] (Figure 2). These findings are primarily from in vitro infection models and require further in vivo validation.

Cross-species evidence supports a potential role for TA systems in persistence-associated phenotypes. In *E. coli*, the SOS-induced TisB toxin lowers proton motive force and ATP levels, promoting persister cell formation [67]. Host stress may induce TA transcription without full toxin activation, maintaining a primed but inactive state [61,68]. Importantly, stress-induced TA transcription does not necessarily indicate toxin activation, as antitoxins can remain bound to toxins, preventing their release [68].

### 4.4. Auxiliary Stress Regulators Contributing to Persistence-Associated Survival

In addition to the stringent response, SOS response, and TA systems, *Salmonella* may further modulate persistence-associated states through several auxiliary regulatory mechanisms. These mechanisms typically interact with core pathways or contribute under specific host-associated conditions.

The RNA-binding protein ProQ influences energy-consuming processes, such as flagella and SPI-2, by regulating bacterial RNA stability and translation, thereby contributing to adaptation to host immune pressure and the maintenance of metabolic balance [69]. Induction of endogenous prophages, such as Gifsy-1, can alter population dynamics through the lysis of a subset of the bacterial population in which the prophage is induced, indirectly affecting the proportion of persister cells. Studies have found that Gifsy-1 induction reduces persister cell formation after ciprofloxacin treatment, suggesting that phage-mediated bacterial lysis may reduce the size of persister-enriched surviving subpopulations after antibiotic exposure [70]. This phenomenon has been demonstrated exclusively in vitro. Whether prophage-mediated lysis similarly limits persister formation during infection remains to be investigated. The sulfur transferase complex may help sustain persistence-associated survival under oxidative stress by regulating thiol homeostasis and redox balance [47].

Another regulatory mechanism involves the small protein MicN, which was recently shown to enhance *Salmonella* antibiotic tolerance and intracellular survival within macrophages. Mechanistically, MicN binds to RpoS and alters its affinity for RNA polymerase, thereby reprogramming transcription and shifting bacterial metabolism toward a low-energy state [71]. Although this study primarily addressed antibiotic tolerance, the MicN-mediated metabolic shift toward a low-energy state may represent a more general mechanism that could also favor entry into persistence-associated states under intracellular conditions.

Furthermore, Hassanin et al. [59] systematically compared the contributions of major stress-response systems to *Salmonella* tolerance and virulence using a series of gene knockout strains. Their results suggest that RpoS, RecA, and RpoE influence multiple stress-associated phenotypes, including biofilm formation, antibiotic tolerance, and intracellular survival, and may also affect persistence-associated behavior under some experimental conditions [59]. Among these, RpoE functions as an envelope stress regulator, and its deletion significantly reduces *Salmonella* survival within macrophages [59], whereas the role of RecA in DNA repair and SOS-associated adaptation is discussed in Section 4.2. Together, these findings suggest that RpoS, RecA, and RpoE respond to nutrient limitation, DNA damage, and envelope stress, respectively, and may contribute to persistence-associated survival under host stress. However, their direct regulatory relationships with one another, as well as with the stringent response, SOS response, and TA systems, remain incompletely resolved. This highlights an important unresolved aspect of the *Salmonella* stress-response network [72].

### 4.5. Integrated Model of Stringent Response–SOS–TA Network Interactions

The stringent response, SOS response, and TA systems all respond to host immune pressure, including acidification, ROS/RNS, and nutrient limitation, and have each been implicated, to varying degrees, in persistence-associated survival under different stress contexts [72]. However, whether these three pathways operate independently or interact in a coordinated manner during infection remains an important unresolved question in the field [32].

Existing studies have revealed several points of interaction among these systems. First, there is bidirectional regulation between the stringent response and TA systems: on one hand, (p)ppGpp has been reported to influence the transcription of various TA genes, and the activation of certain TA modules (e.g., *ghoST*, *relBE*, and *ryeA*) has been implicated in stress adaptation [73]. On the other hand, some TA systems can feedback on stringent response signaling. For example, toxic small alarmone synthetase (toxSAS) family toxins themselves have (p)ppGpp synthase activity and can directly participate in the production of (p)ppGpp [74], while mRNA endonuclease (mRNase) toxins can be rapidly activated under stress, degrade mRNA, and provide negative feedback on (p)ppGpp levels, thereby preventing excessive activation of the stringent response [75] (Figure 2).

Second, the SOS response and TA systems exhibit functional overlap. In addition to repairing DNA damage, the SOS response has been linked to persistence regulation in model bacteria through induction of effectors such as TisB [57], which provides a conceptual example of overlap between DNA-damage signaling and growth-arrest programs. Notably, although TisB is induced by the SOS response, its mechanism of action, namely ATP depletion, is similar to that of canonical TA toxins, suggesting an interaction between these two pathways at the effector level [57,67] (Figure 2). In contrast, direct evidence for interactions between the stringent response and the SOS response remains limited.

At the effector level, these three pathways share a common outcome in that they can promote bacterial growth restriction through distinct mechanisms: the stringent response reduces growth by reprogramming global metabolism, the SOS response may support persistence-associated survival by maintaining DNA integrity under stress, and TA systems can directly inhibit proliferation by blocking translation or lowering ATP availability. Although the extent of their interaction remains incompletely resolved, these pathways may converge at the level of reduced metabolic activity, growth arrest, and altered ATP homeostasis, thereby contributing to both population-level tolerance and subpopulation-level persistence under host pressure, depending on the degree of pathway activation and the specific niche context [12,46] (Figure 2).

## 5. Host Microenvironments and the Bidirectional Shaping of *Salmonella* Persistence

**Figure 3:**
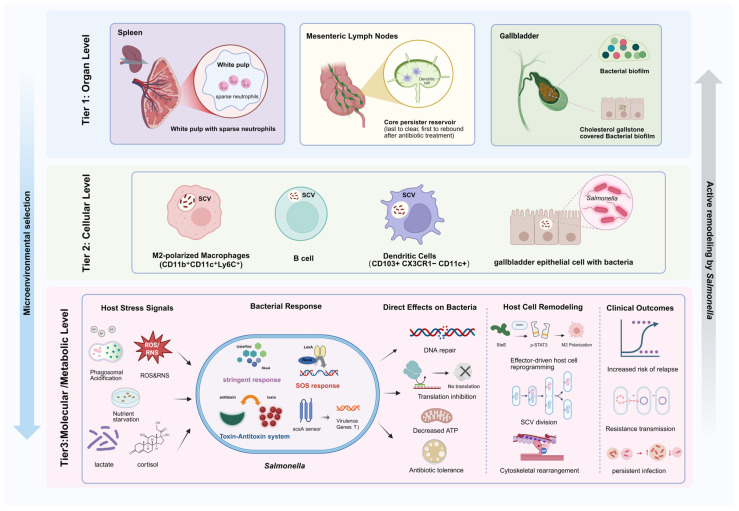
Multiscale host niches shaping persistence-associated *Salmonella* survival. This schematic illustrates how host microenvironments shape *Salmonella* persistence across three interconnected levels. Organ level: Distinct anatomical niches support tissue-specific persistence; the splenic white pulp, characterized by low neutrophil density, may function as an immunological sanctuary, mesenteric lymph nodes (MLNs) can remain colonized after antibiotic treatment and serve as persistence reservoirs, and the gallbladder supports persistence through biofilm formation on cholesterol gallstones and epithelial cell invasion. Cellular level: Persistence-associated subpopulations are enriched in specific host cell types, with M2-polarized macrophages and B cells harboring non-replicating bacteria in the spleen, while CD103^+^ dendritic cells in MLNs contain metabolically quiescent *Salmonella*. Molecular/metabolic level: Host-derived stress signals—including phagosomal acidification, ROS/RNS, nutrient limitation, lactate, and cortisol—activate bacterial stress-response pathways, with the stringent response, SOS response, and toxin–antitoxin systems promoting metabolic remodeling, growth arrest, and stress survival. Collectively, these processes contribute to reduced growth and antibiotic tolerance while supporting persistence within host cells and tissue niches. Blue arrows indicate microenvironmental selection, and gray arrows represent active bacterial remodeling. BioRender Inc. (Toronto, ON, Canada). https://BioRender.com (accessed on 23 March 2026).

The molecular mechanisms by which *Salmonella* enters persistence-associated states confer the potential for survival under stress. However, whether this potential is realized in vivo depends on the specific microenvironment inhabited by the bacteria [14]. The anatomical structures of different tissues and organs, the polarization state of immune cells, and the differential concentrations of local metabolites together create spatially distinct selective pressures on persistence-associated phenotypes [63,76]. This section, framed within the concept of “spatial geography,” systematically explains how the host microenvironment selects and maintains diverse persistence-associated bacterial phenotypes, and how *Salmonella* actively reshapes the immune microenvironment to establish its own persistent ecological niche. This multi-scale interaction may ultimately reflect a bet-hedging strategy for bacterial populations, enabling long-term survival in complex host environments [22] (Figure 3).

### 5.1. Organ-Level Spatial Heterogeneity and Tissue Reservoirs

Single-cell studies in experimental infection models have revealed that even within host tissues, bacterial physiological states exhibit considerable heterogeneity. Using a mouse model of *Salmonella* infection, Claudi et al. employed a fluorescent growth rate reporter to show that within infected tissues, *Salmonella* exists in different subpopulations, including rapidly proliferating and slowly growing cells. They found that the bactericidal effect of antibiotics is closely related to the single-cell division rate of the bacteria [35]. Whether such heterogeneity directly translates to human infection remains to be determined.

The spatiotemporal differences in the host immune response also contribute to the generation of this heterogeneity. Recent scIVNL-seq technology, with single-cell resolution, has revealed that after *Salmonella* infection, the activation of immune cells residing in different tissues (such as bone marrow macrophages and intestinal CD8^+^ T cells) exhibits significant differences in time, space, and transcriptional dynamics [77].

The distribution of *Salmonella* in the body and its persistence are influenced by the host’s local microanatomy. In the splenic white pulp, low neutrophil density and limited immune clearance allow *Salmonella* to survive antibiotic treatment and form a sanctuary [78] (Figure 3). This difference in the local immune effector cell distribution may lead to the coexistence of persistence-associated subpopulations and actively replicating bacteria within the same organ. The mesenteric lymph nodes (MLNs) are the last site to maintain *Salmonella* colonization after antibiotic treatment and the first to restore bacterial activity after antibiotic withdrawal, highlighting their special role as a potential core reservoir for persistence [79].

The gallbladder is a well-recognized reservoir for chronic carriage and relapse of typhoidal *Salmonella* and plays an important role in long-term colonization and persistence. On the one hand, cholesterol gallstones provide a unique extracellular niche for *Salmonella* persistence. In a mouse model fed a stone-inducing diet, biofilm formation on gallstones significantly enhanced bacterial colonization in gallbladder tissue and bile, and increased fecal shedding [80] (Figure 3).

On the other hand, the gallbladder epithelium itself provides an intracellular niche for *Salmonella* persistence. Gonzalez-Escobedo et al. found that *Salmonella* can invade polarized gallbladder epithelial cells in an SPI-1-dependent manner, replicate intracellularly, and form microcolonies. In a chronic infection mouse model, bacterial microcolonies were still observed on the gallbladder epithelium surface 21 days post-infection, with the infection site exhibiting abundant macrophage infiltration and relatively few neutrophils [81]. The biofilms on the surface of gallstones, together with intracellular persistence in gallbladder epithelial cells, form a comprehensive picture of the gallbladder reservoir.

Studies using gene-tagged strains, including wild-type isogenic tagged strains (WITS), combined with mathematical models have further revealed that although bacteria undergo rapid replication during the early stages of infection, this is accompanied by a high mortality rate. Bacteria form independent and randomly established subpopulations in different organs, and this process is tightly regulated by the host’s antimicrobial defenses [82]. Consistent with this compartmentalized view, Li et al. demonstrated in a mouse model that the splenic white pulp, characterized by low neutrophil density, serves as an immunological sanctuary where *Salmonella* survives antibiotic treatment, providing direct in vivo evidence that tissue microanatomy can shape bacterial persistence during chemotherapy [78]. Together, these observations support the idea that bacterial fate is strongly influenced by the tissue niche in which the bacteria reside, consistent with the “spatial geography” framework.

### 5.2. Cellular Niches for Persistence-Associated Subpopulations

At the cellular level, Helaine et al. used fluorescence dilution to track intracellular bacterial replication at the single-cell level. They found that a substantial fraction of *Salmonella* entered a non-replicating state within macrophages under acidic, nutrient-limited, and oxidative stress conditions. This suggests that macrophages may represent a potential niche for persistence-associated subpopulations in experimental models [29]. After antibiotic treatment in experimental models, *Salmonella* was reported to be enriched in CD103^+^CX_3_CR1^−^CD11c^+^ dendritic cells within the cecum-draining lymph nodes, where it enters a low-metabolism state and exhibits phenotypic tolerance to fluoroquinolones [83] (Figure 3).

The liver has also been implicated as a site associated with chronic infection in experimental settings and is characterized by an immune environment enriched in regulatory CD4^+^ T cells and M2-like macrophages, which may favor long-term *Salmonella* persistence [76]. This selective colonization may help the bacteria evade host immune clearance. In splenic granulomas, *Salmonella* tends to persist in M2-polarized macrophages with enhanced fatty acid oxidation, and CD11b^+^CD11c^+^Ly6C^+^ macrophages dominate in human granulomas, where *Salmonella* can survive long-term [84] (Figure 3).

Recent studies have further expanded the range of host cell types capable of harboring persistence-associated bacteria to include B cells. Cruz-Cruz et al. reported that splenic B cells harbor non-replicating, antibiotic-tolerant *Salmonella* in experimental models. Similar to persistent bacteria in other immune cells, *Salmonella* in B cells is also in a non-replicating state and exhibits antibiotic tolerance to cefotaxime and ciprofloxacin. Notably, the formation of this persistence-associated phenotype depends on the SehA/B and RelE/B TA systems, rather than the classical SPI-2 type III secretion system (T3SS) [85] (Figure 3).

Nutrient limitation, as a host-imposed metabolic constraint that can contribute to persistence-associated states, is widely present in various non-macrophage host cell types. In human epithelial cells, *Salmonella* can enter a dormant-like state to survive long-term [86]. In fibroblasts, non-replicating *Salmonella* can utilize different SPI-2 effector proteins to achieve long-term survival [87]. Beyond cellular polarization, nutritional immunity within these niches also drives heterogeneity. For instance, Roche et al. recently provided in vivo evidence that a subset of *Salmonella* exploits erythrophagocytosis in mice to access iron-rich endosomes within macrophages, thereby bypassing SLC11A1-imposed iron deprivation and sustaining a persistence-competent state [30]. These findings expand the spectrum of cellular niches in which persister bacteria may reside.

### 5.3. Host Metabolic Cues and Stress Signals Regulating Persistence Dynamics

The selective preferences and active remodeling at the cellular level described above are closely linked to the overall metabolic state of the host. In the infectious microenvironment, hypoxia and inflammation-driven glycolysis lead to the accumulation of lactic acid at the infection site. Wang et al. found that macrophage-derived lactic acid promotes M2 polarization in a SteE-dependent manner. Specifically, lactic acid upregulates SteE expression and enhances its function, thereby promoting macrophage polarization toward an M2-like state. This may indirectly support the intracellular survival and replication of *Salmonella* [88] (Figure 3).

In polarized M2 macrophages, the host transcription factor PPARδ is significantly upregulated. Using a mouse chronic infection model, Eisele et al. found that PPARδ, a key regulator of lipid metabolism, activates fatty acid oxidation-related genes, thereby reshaping host cell metabolism. This process not only provides energy to host cells but also increases intracellular glucose availability. This metabolic reprogramming supplies carbon sources and energy that support *Salmonella* survival and long-term persistence within macrophages. Functional studies confirmed its necessity—PPARδ-deficient mice failed to sustain chronic infection, whereas pharmacological PPARδ activation enhanced bacterial intracellular survival and replication [89].

Additionally, the host’s systemic stress response plays a role in regulating persistence. Verbrugghe et al. found that when the host is in a stressed state, levels of glucocorticoids, such as cortisol, rise. These signaling molecules can be sensed by *Salmonella* through the ScsA-associated pathway. This sensing mechanism triggers bacterial adaptive responses, with ScsA regulating the expression of virulence genes within macrophages, promoting bacterial proliferation and stimulating host cell cytoskeletal rearrangement. Together, these findings suggest that host stress signals may influence intracellular adaptation and persistence-related reactivation by regulating bacterial responses within host cells [90] (Figure 3).

Host immune signals not only influence the formation of persistence-associated states but may also regulate the reactivation of persister bacteria. Ronneau et al. found that host RNS can lock persister bacteria in a growth-arrested state by interfering with the bacterial TCA cycle. When RNS levels decrease during the later stages of infection, these persister bacteria resume growth in a slow and highly heterogeneous manner. This mechanism helps explain how persistence-associated reservoirs may continue to contribute to infection recurrence after antibiotic withdrawal [45].

### 5.4. Salmonella-Mediated Remodeling of Immune Niches

*Salmonella* actively remodels the immune microenvironment through virulence-associated host reprogramming, thereby constructing niches that may be permissive for persistence-associated survival. Studies have shown that intracellular persister *Salmonella* retain metabolic activity and can secrete effector proteins via the SPI-2 T3SS to suppress pro-inflammatory responses and induce M2 polarization [91]. Hill et al. discovered, by comparing the physiological differences between tolerant and persister bacteria, that unlike tolerant bacteria, which enter a nearly dormant state, persister cells retain “fragile diversity”: they maintain an active metabolic state but are highly susceptible to DNA double-strand breaks induced by macrophages. If DNA repair mediated by RecA occurs, infection relapse may be promoted [22]. Schulte et al. confirmed that most intracellular non-replicating persisters exhibit metabolic activity yet display reduced stress responses, suggesting the existence of a “protected physiological niche” that enables environmental sensing while minimizing host stress [8].

A key mechanism driving M2 polarization is the T3SS effector SteE. Panagi et al. revealed that SteE is phosphorylated by host glycogen synthase kinase 3 (GSK3). This phosphorylation converts GSK3 into an atypical tyrosine kinase that directly phosphorylates signal transducer and activator of transcription 3 (STAT3) at Tyr705. Consequently, STAT3-dependent anti-inflammatory gene expression is driven independently of the Janus kinase (JAK) pathway [92,93]. In a mouse splenic granuloma model, Pham et al. demonstrated that SteE activity drives M2 polarization, while host TNF signaling counteracts this process; TNF neutralization enhanced M2 polarization and bacterial persistence in a SteE-dependent manner [84] (Figure 3). This dynamic interplay between TNF and SteE shapes macrophage polarization and the fate of persister bacteria.

Leiba et al. utilized a zebrafish model and high-resolution real-time imaging technology to reveal dynamic changes in macrophage phenotypes during the course of infection in vivo. In the early stages of infection, macrophages exhibit an M1 pro-inflammatory phenotype, restricting bacterial proliferation. In the later stages of infection, *Salmonella* persists within stationary macrophages that transition to an anti-inflammatory/regenerative state. Transcriptomic analysis further confirmed that these macrophages undergo a shift from a pro-inflammatory to an anti-inflammatory/regenerative phenotype, accompanied by changes in the expression of adhesion-related genes [33].

### 5.5. Bet-Hedging and the Evolutionary Significance of Phenotypic Heterogeneity

The phenotypic heterogeneity shaped by the host microenvironment at both the spatial and molecular levels may reflect a bet-hedging strategy employed by bacterial populations to cope with the complex and dynamic host environment [94]. This strategy may allow bacterial populations to maintain subpopulations adapted to specific microenvironments even under strong antibiotic pressure and immune clearance, thereby contributing to persistence and infection recurrence.

The formation of persistence-associated heterogeneity involves the coupling of metabolic regulation and global transcriptional regulation. For instance, the nucleotide-related regulator Fis modulates the proportion of persisters via glutamate metabolism [95], illustrating how metabolic–transcriptional coupling may translate host signals into a persistence-associated phenotype.

## 6. Clinical and Translational Implications of *Salmonella* Persistence

### 6.1. Persistent Reservoirs and Endogenous Relapse

**Figure 4:**
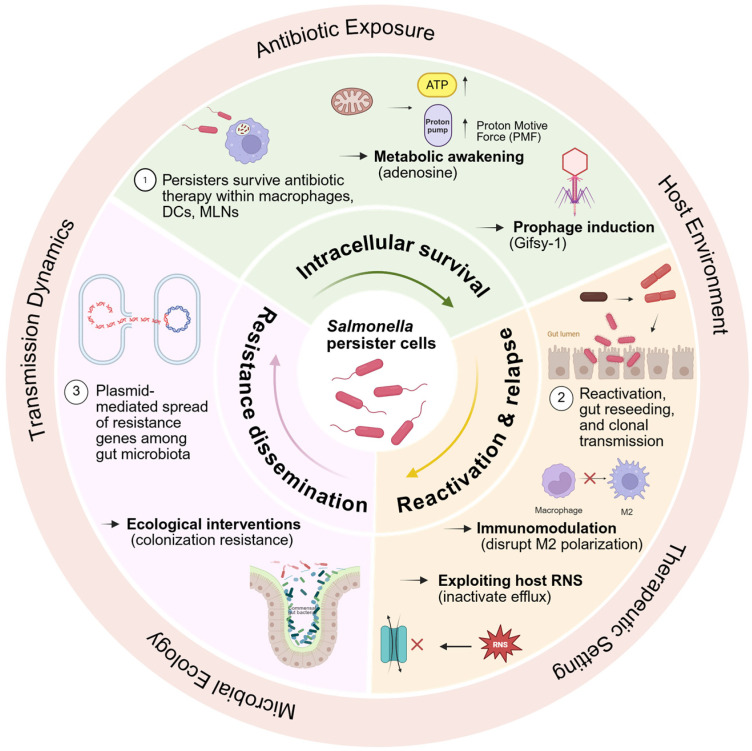
Clinical consequences and emerging intervention strategies targeting *Salmonella* persistence. Intracellular survival during antibiotic therapy is targeted by metabolic awakening and prophage induction; reactivation and relapse is addressed by immunomodulation and host reactive nitrogen species (RNS); and resistance dissemination via horizontal gene transfer (HGT) is countered by ecological interventions restoring colonization resistance. The outer circle indicates key contextual factors including antibiotic exposure, transmission dynamics, microbial ecology, and therapeutic settings. BioRender Inc. (Toronto, ON, Canada). https://BioRender.com (accessed on 2 May 2026).

Persister cells and persistence-associated bacterial subpopulations can contribute to the formation of long-term latent reservoirs in host tissues, providing a potential mechanistic basis for infection relapse. Using fluorescent single-cell analysis, Helaine et al. demonstrated that upon internalization by macrophages, *Salmonella* rapidly forms a nonreplicating persister subpopulation induced by vacuolar acidification and nutritional deprivation. Notably, some persisters resume intracellular growth after phagocytosis by naïve macrophages, establishing a reservoir that can seed recurrent infection [24]. This phenotypic heterogeneity—where a subpopulation enters a slow-growing or growth-arrested state—is a hallmark of persistence. Such cells can survive lethal antibiotic exposure without genetic resistance and resume growth after stress removal, contributing to the recalcitrance and relapse of persistent infections [7]. Tissues such as the mesenteric lymph nodes serve as anatomical niches where *Salmonella* persists in dormant or low-metabolism states. After antibiotic cessation, these reservoirs can reactivate, potentially contributing to recurrent infection [96]. Using a mouse model, Newson et al. recently demonstrated that *Salmonella* forms antibiotic-recalcitrant reservoirs in host tissues. After therapy cessation, these reservoirs reactivate, reseeding the gut lumen and leading to clonal transmission [23] (Figure 4).

### 6.2. Persistence-Associated Survival and Resistance Evolution

Persister cells and persistence-associated subpopulations may contribute to antibiotic resistance evolution in vivo in two related ways. First, by surviving antibiotic treatment and host-derived stresses, these cells can prolong bacterial survival during infection. This provides a temporal window in which resistance-conferring mutations may arise and become enriched under continued selective pressure [12,16]. Second, by enabling reseeding of luminal compartments, persistence-associated reservoirs increase opportunities for contact with resident microbiota, creating ecological conditions permissive for horizontal gene transfer. For example, tissue-resident *Salmonella* can reseed the gut lumen, where plasmid transfer to resident enteric bacteria occurs efficiently [23,97]. In addition, mobile genetic elements such as the pESI megaplasmid can co-localize antimicrobial resistance determinants with genes implicated in stress adaptation or persistence-associated phenotypes, raising the possibility of co-selection under host and antibiotic pressures [98]. Together, these observations support the more cautious view that persistence-associated survival may create ecological and temporal conditions that favor the emergence or enrichment of resistant mutants, and may increase opportunities for horizontal gene transfer in host-associated environments. However, direct causal links remain incompletely defined (Figure 4).

### 6.3. Emerging Anti-Persistence Strategies

The concept that some persistence-associated subpopulations remain metabolically active despite growth arrest has motivated “metabolic awakening” strategies in experimental systems. In this context, exogenous adenosine (ADO) has been reported to increase bacterial ATP availability and proton motive force through purine salvage–linked metabolic activation, thereby enhancing antibiotic uptake in otherwise tolerant subpopulations [99,100]. Other strategies have been explored to more directly reduce persistence. Induction of prophage lytic cycles reduces persister formation in vitro [70]. Immunomodulation that interferes with *Salmonella*-driven M2-like macrophage polarization can reduce the permissiveness of intracellular niches [84]. Host-derived reactive nitrogen species (RNS) constrain bacterial metabolism and reduce efflux-associated tolerance, potentially enhancing intracellular antibiotic activity [101].

Ecological interventions that restore colonization resistance may limit post-treatment luminal re-expansion of *Salmonella* and reduce opportunities for resistance gene exchange [23]. Overall, these strategies remain emerging or conceptual rather than clinically established (Figure 4).

Beyond clinical settings, *Salmonella* persistence also poses significant challenges in food production and animal husbandry. Persistent *Salmonella* can asymptomatically colonize livestock, particularly poultry, and survive routine sanitation procedures in food processing environments, thereby serving as reservoirs for zoonotic transmission [98,102]. In these settings, persister cells may contribute to the failure of eradication programs and facilitate the dissemination of resistant strains through the food chain [103]. Future control strategies should therefore integrate interventions targeting persistence across both human and animal systems within a One Health framework.

## 7. Conclusions and Outlook

The persistence-associated state of *Salmonella* is an adaptive physiological state that emerges from dynamic host–pathogen interactions. This state is not simply a form of growth arrest, but is shaped by host microenvironmental pressures and may stabilize a metabolically active, non-replicating state under multiple selective constraints. This enables bacteria to survive strong immune responses and antibiotic pressure, thereby contributing to infection relapse, immune evasion, and persistence-associated survival under antimicrobial stress. The persistent state is best understood as a dynamic, heterogeneous, and ecologically functional spectrum of physiological states, rather than a single static subpopulation. The persistence-associated state may also provide an important link between phenotypic tolerance and the evolution of genetic resistance by creating a temporal window for the emergence and enrichment of resistance-conferring mutations. This may increase within-host opportunities for the dissemination of resistance determinants and, under some conditions, may also contribute to onward transmission.

Despite significant progress in this field, research on *Salmonella* persistence still faces several key challenges:

(1) Limitations of current research models and challenges in quantifying in vivo heterogeneity: Studies on the persistence-associated state remain highly dependent on experimental systems, and there is still a lack of techniques that can dynamically and quantitatively distinguish population-level tolerance from persister subpopulations in vivo.
(2) Insufficient systematic characterization of persister heterogeneity, which limits the transition from population-level observations to single-cell resolution: Recent advances in single-cell technologies have identified candidate physiological signatures of persister cells—including non-replicating behavior, low ATP levels, and reduced stress responses—and have demonstrated that the persistence-associated state is dynamic and reversible. However, these characteristics should currently be regarded as correlative rather than definitive biomarkers. Identifying distinct persister subpopulations within the broader heterogeneous population and developing specific markers for their reliable detection therefore remain major challenges in the field.
(3) Challenges in translating experimental anti-persistence strategies into clinical practice: Several anti-persistence strategies have shown promise under experimental conditions, including metabolic awakening, phage induction, and modulation of the host immune microenvironment. However, their safety, specificity, and potential synergy with conventional antibiotics still require careful validation before clinical application.

In the face of these challenges, future intervention strategies may need to move beyond traditional bactericidal approaches and toward multi-target, system-level ecological interventions: targeting metabolic vulnerabilities by using “metabolic awakening” to reverse the low-energy state of persistence-associated subpopulations; reshaping the immune microenvironment to disrupt the immune-evasion niche established by persisting bacteria; and limiting transmission-associated ecological opportunities to reduce the spread of resistance determinants. Understanding the persistent state of *Salmonella* at the intersection of host microenvironment, immune regulation, and microbial ecology not only provides a broader perspective for reassessing the causes of antibiotic treatment failure, but also offers a conceptual framework for the development of next-generation intervention strategies against chronic infection and resistance evolution.

## Data Availability

No new data were created or analyzed in this study. Data sharing is not applicable to this article.

## Abbreviations

The following abbreviations are used in this manuscript:

NTS	non-typhoidal *Salmonella*
CDC	Centers for Disease Control and Prevention
MIC	minimum inhibitory concentration
MDK	minimum duration of killing
HGT	horizontal gene transfer
PAP	population analysis profile
VBNC	viable but non-culturable
ROS	reactive oxygen species
RNS	reactive nitrogen species
NAD^+^	nicotinamide adenine dinucleotide
NADH	reduced form of NAD^+^
TCA	tricarboxylic acid
SPI-1	*Salmonella* pathogenicity island 1
SPI-2	*Salmonella* pathogenicity island 2
iNOS	inducible nitric oxide synthase
(p)ppGpp	guanosine tetraphosphate and guanosine pentaphosphate
TA	toxin–antitoxin
MLNs	mesenteric lymph nodes
WITS	wild-type isogenic tagged strains
T3SS	type III secretion system
ADO	adenosine
GSK3	glycogen synthase kinase 3
STAT3	signal transducer and activator of transcription 3
JAK	Janus kinase
PMF	proton motive force
DCs	dendritic cells
TacT	tRNA acetyltransferase toxin
